# Bilateral Peritonsillar Abscess Secondary to Infectious Mononucleosis

**DOI:** 10.7759/cureus.92958

**Published:** 2025-09-22

**Authors:** Athanasios Vlachodimitropoulos, Alkmini Gatsounia, Gerasimos Danielides, Christine Dafni, Spyridon Lygeros

**Affiliations:** 1 Otolaryngology - Head and Neck Surgery, University General Hospital of Patras, Patras, GRC; 2 Department of Pharmacy, University Hospital of Ioannina, Ioannina, GRC

**Keywords:** ebv complications, epstein-barr, infectious mononucleosis, peritonsillar abscess, tonsillitis, trismus

## Abstract

Infectious mononucleosis (IM) is a clinical entity caused by Epstein-Barr infection. Serious complications during the acute phase of the infection are rare, and very few cases with bilateral peritonsillar abscesses (PTAs) have been reported to date. A 23-year-old man presented to our emergency department with fever, dysphagia, and trismus that were unresponsive to prescribed antibiotics. Physical examination revealed a bilateral PTA. The patient was admitted to the otolaryngology department, where surgical drainage of both abscesses was performed by incision. The diagnosis of bilateral PTA can be challenging because it lacks the typical clinical manifestations of unilateral PTA. Although CT imaging is frequently performed, it is not essential for diagnosis, as physical examination alone should raise suspicion, particularly in primary care settings. The combination of surgical drainage, appropriate antibiotic therapy, and close follow-up is the cornerstone of treatment. This case highlights that even common infections like IM can give rise to rare and potentially serious complications, underscoring the importance of maintaining a high index of suspicion to ensure timely diagnosis and management.

## Introduction

Infectious mononucleosis (IM) is a clinical syndrome caused primarily by the Epstein-Barr virus (EBV), characterized by pharyngitis, cervical lymphadenopathy, malaise, and fever. It occurs predominantly in adolescents and young adults and is usually transmitted through oropharyngeal secretions, hence the popular designation “the kissing disease” [1]. In most cases, the disease follows a benign and self-limiting course, with severe complications during the acute phase being uncommon [1]. When complications do occur, they more often involve hematologic, hepatic, or neurologic systems, while significant upper airway infections are rare [1,2].

Peritonsillar abscess (PTA) is the most frequent deep neck space infection, typically arising as a sequela of acute tonsillitis and most often presenting unilaterally [2]. PTA associated with IM is uncommon, and bilateral involvement is exceedingly rare, with only three cases reported in the English medical literature to date [3]. This unusual presentation is clinically significant because of the potential for rapid airway compromise, underscoring the need for timely recognition and intervention. Here, we describe a case of severe IM complicated by bilateral PTA.

## Case presentation

A 23-year-old man was referred to our emergency department with a five-day history of fever (maximum 39°C), sore throat, dysphagia, and fatigue. He was treated with oral clarithromycin (500 mg twice daily) for three days without improvement. His past medical history was unremarkable.

On admission, his temperature was 38.5°C, while the remaining vital signs were within normal limits. Physical examination revealed a muffled voice, marked trismus, bilateral cervical lymphadenopathy, and severe bilateral tonsillar enlargement with the tonsils in midline contact (“kissing tonsils”), covered by exudate. The uvula was mildly edematous but not deviated. His complete blood count was 12,580/μL leukocytes with 58.9% neutrophils, 31.8% lymphocytes, and 8.5% monocytes. C-reactive protein level was 27.37 mg/dL, and liver enzymes were within the normal range. Serological tests were positive for EBV VCA IgM and IgG. On this basis, IM was considered the most likely diagnosis.

**Figure 1:**
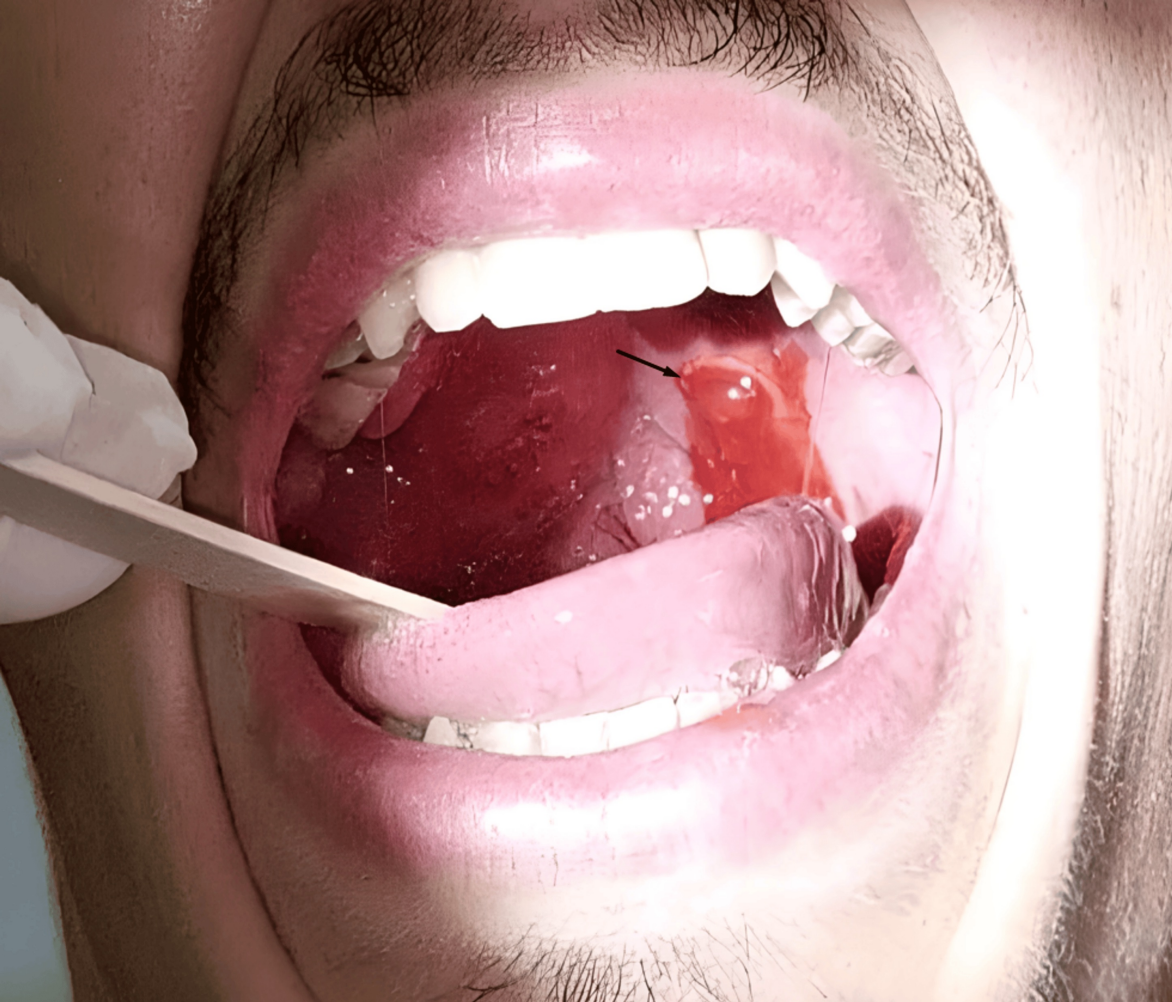
Drainage of the left peritonsillar abscess (arrow) after positive aspiration

**Figure 2:**
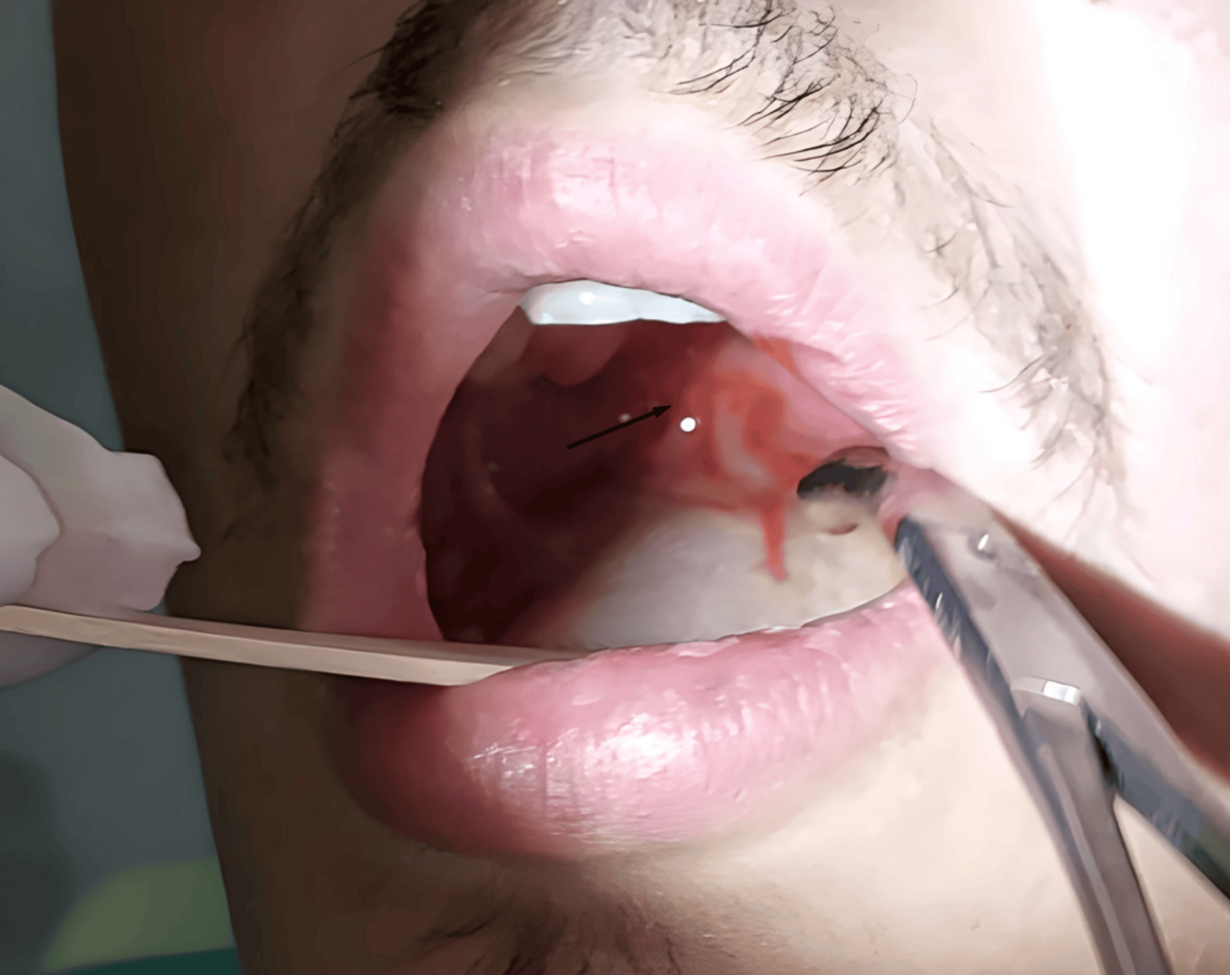
Subsequent drainage of the right peritonsillar abscess (arrow) after positive aspiration

Because of trismus and edema of both anterior peritonsillar pillars, diagnostic needle aspiration was performed bilaterally for suspected PTA. Aspiration was positive on each side, and incisions with drainage were successfully performed (Figures 1-2). Flexible nasolaryngoscopy was normal, revealing no airway compromise. The patient was admitted for intravenous clindamycin therapy, hydration, analgesia, and close monitoring.

**Figure 3:**
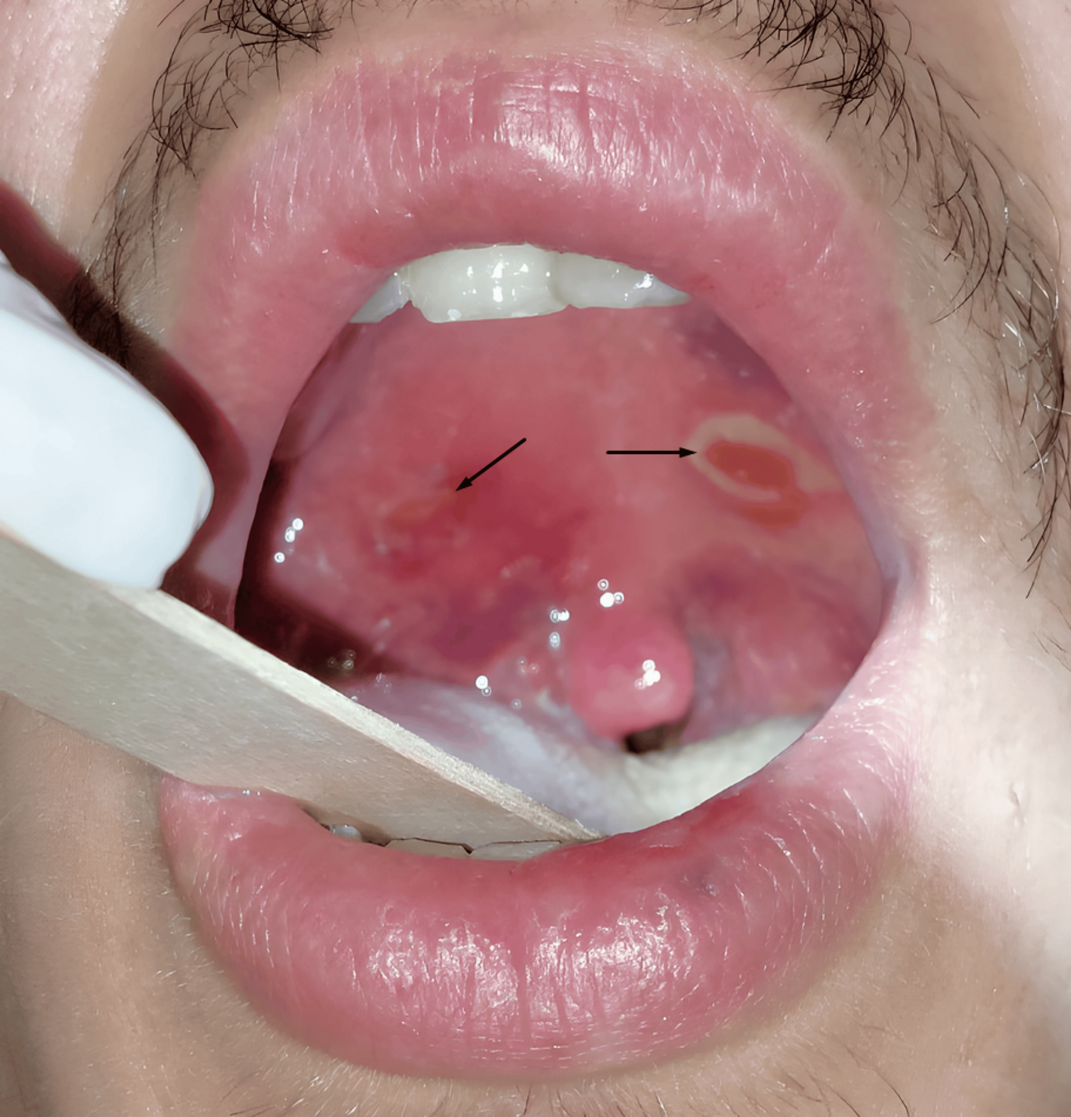
Oral view of the incision sites (arrows) after total evacuation of the abscess cavities

Over the following days, repeated bilateral drainage was performed, with progressively decreasing amounts of pus being drained (Figure 3). The patient’s clinical condition and laboratory parameters improved significantly. Despite negative cultures obtained separately from both abscesses, antibiotic therapy was continued. He was discharged on hospital day 4 with a 10-day course of oral clindamycin.

## Discussion

IM is common in clinical practice and can be associated with a wide range of complications such as upper airway obstruction, hepatosplenomegaly, and hemolytic anemia [1]. PTA is a rare complication, occurring in approximately 1% of the patients admitted with a diagnosis of IM [2]. Cases of bilateral PTA are even rarer [3].

The peritonsillar space is defined as the space between the capsule of the palatine tonsil and the superior constrictor muscle of the pharynx [4]. PTA is regarded as the final stage of a disease continuum that begins with acute exudative tonsillitis, progresses to cellulitis, and ultimately results in the formation of an abscess [5]. PTAs are the most common deep neck infections and often arise as a complication of acute tonsillitis [5]. While unilateral PTA is relatively common, bilateral involvement is rare, with an overall reported incidence of 4.9% [6]. However, the low incidence of bilateral PTA may be due to early diagnosis and treatment with antibiotics prior to progression to the contralateral site or underreporting [5].

In IM, the inflamed pharynx and necrotic tonsils can be complicated by bacterial superinfection such as hemolytic *Streptococcus* (Lancefield groups A, C, and G), *Fusobacterium necrophorum*, and *Staphylococcus aureus* [7,8]. During acute EBV infection, there is decreased mucosal production of antibodies that could allow bacterial attachment to the tonsillar epithelium with subsequent bacterial penetration, resulting in abscess formation [3,9]. However, EBV infection alone appears to cause suppurative complications involving the tonsils, with plausibly a higher frequency of bilateral PTA than in bacterial tonsillitis [10]. In our case, the culture results were negative, but this could be attributed to the patient’s prior antibiotic exposure (clarithromycin) or to the fastidious nature of anaerobic organisms commonly involved in PTA. However, considering that the vast majority (>70%) of PTA are polymicrobial with aerobic and anaerobic organisms, we decided to administer clindamycin as part of our treatment plan [11].

The diagnosis of PTA is essentially clinical. PTA generally presents with a muffled voice, progressive odynophagia, referred otalgia, trismus, drooling, and fever [3]. In the vast majority of unilateral cases, patients present with tonsillar and palatal asymmetry, uvular deviation, and ipsilateral otalgia. The difficulty in the diagnosis of bilateral PTA is the absence of these typical signs [5].

CT imaging can help diagnose bilateral PTA and should be considered when there is significant trismus but no unilateral inflammatory findings are present [12]. It can also effectively differentiate PTA from peritonsillar cellulitis [13]. However, contrast-enhanced CT is not always available, particularly in the primary care setting, where the majority of cases of IM are seen and treated. In our case, a high index of suspicion enabled us to diagnose and treat our patient effectively. CT imaging was not required because our patient showed significant improvement shortly after bilateral drainage and was afebrile within 24 hours. He showed no signs of sepsis, had a patent airway on endoscopy, and was not likely to have further deep neck involvement. However, bilateral PTAs are associated with a higher risk of further complications such as airway obstruction and extension into deeper neck compartments [5]. In these situations, CT imaging or even MRI should be strongly considered.

Treatment of PTA remains controversial. The main options are needle aspiration, incision and drainage, and immediate tonsillectomy combined with antibiotic therapy. Immediate tonsillectomy is a straightforward surgical procedure that quickly alleviates trismus and achieves complete drainage by excising the entire medial wall of the abscess cavity [6]. Such an operation may subsequently reveal an unsuspected contralateral PTA [14]. However, it is neither the most cost-effective solution nor without risk, as tonsillectomy can be associated with intraoperative and postoperative hemorrhage, as well as anesthetic complications, particularly in patients with underlying health problems [3]. When combined with antibiotic therapy, drainage, regardless of the method used, leads to abscess resolution in over 90% of cases [15]. Most authors agree that for the majority of PTAs, both needle aspiration and incision drainage, alone or in combination, are the mainstay of management. However, they often result in incomplete drainage of the cavity and may require several repetitions, which can cause discomfort to the patient. In addition, if interval tonsillectomy is planned, the procedure can be technically challenging due to the fibrosis that develops around the tonsil [14,15]. In our clinic, a diagnostic needle aspiration followed by incision drainage is performed with intravenous antibiotic administration and repeated evacuation of the cavity as needed. This was the first case of bilateral PTA to present to our department, and it was successfully managed using the approach described above.

## Conclusions

Although unilateral PTA is the most common suppurative complication of acute tonsillitis, bilateral involvement is uncommon. Clinicians should remain vigilant for this rare but clinically significant complication of IM, as early recognition and intervention are crucial for optimal patient outcomes. While physical examination remains of paramount importance, particularly in primary care settings where access to imaging may be limited, imaging techniques can provide valuable support in complex cases by confirming the diagnosis and identifying additional complications. A high index of suspicion, timely surgical drainage, and appropriate antimicrobial therapy are crucial in preventing airway compromise.
